# Supplementary material for: Femtosecond-precision electronic clock distribution in CMOS chips by injecting frequency comb-extracted photocurrent pulses
Source: Nat Commun. 2023 Apr 24;14:2345. doi: 10.1038/s41467-023-38122-3 (PMC10125959; doi:10.1038/s41467-023-38122-3)
Supplement: Supplementary file 1 — Supplementary Information [file 41467_2023_38122_MOESM1_ESM.pdf]

Supplementary Information for

Femtosecond-precision electronic clock distribution in CMOS chips by injecting frequency comb-extracted photocurrent pulses

Minji Hyun, Hayun Chung, Woongdae Na, and Jungwon Kim

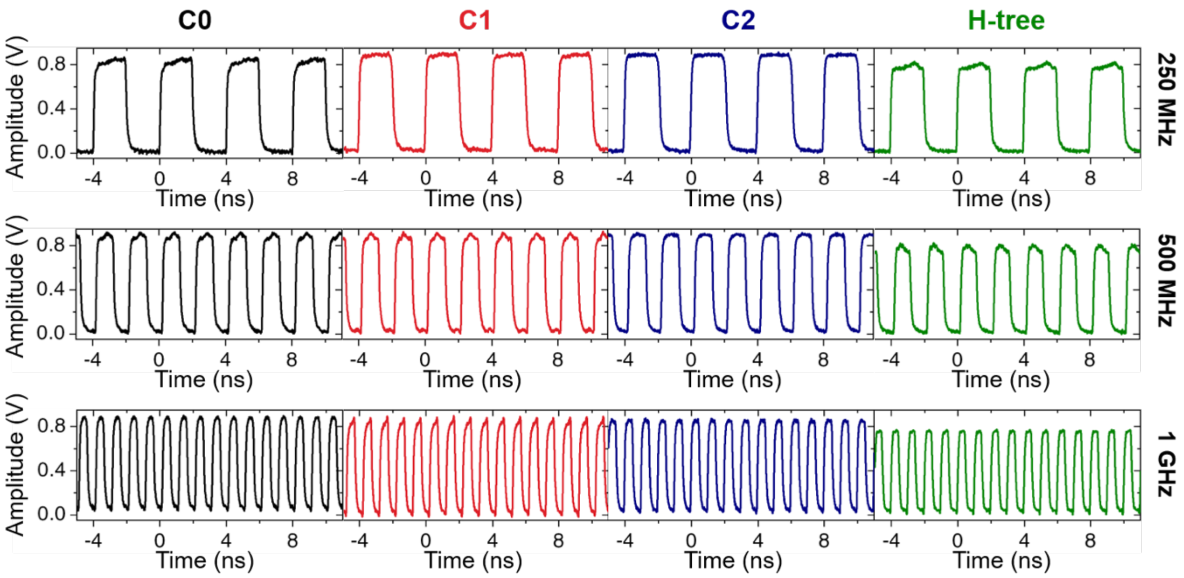

Supplementary Fig. 1. Clock waveforms of each clock domain for different clock rates. Output clock waveforms from each clock domain at clock rates of 250 MHz, 500 MHz, and 1 GHz. Shown waveforms were taken when dynamic clock loads in C1 and C2 were turned on and H-tree structure CDN was also operating. Note that there was no noticeable change of waveforms between the conditions when dynamic loads were turned on and off.

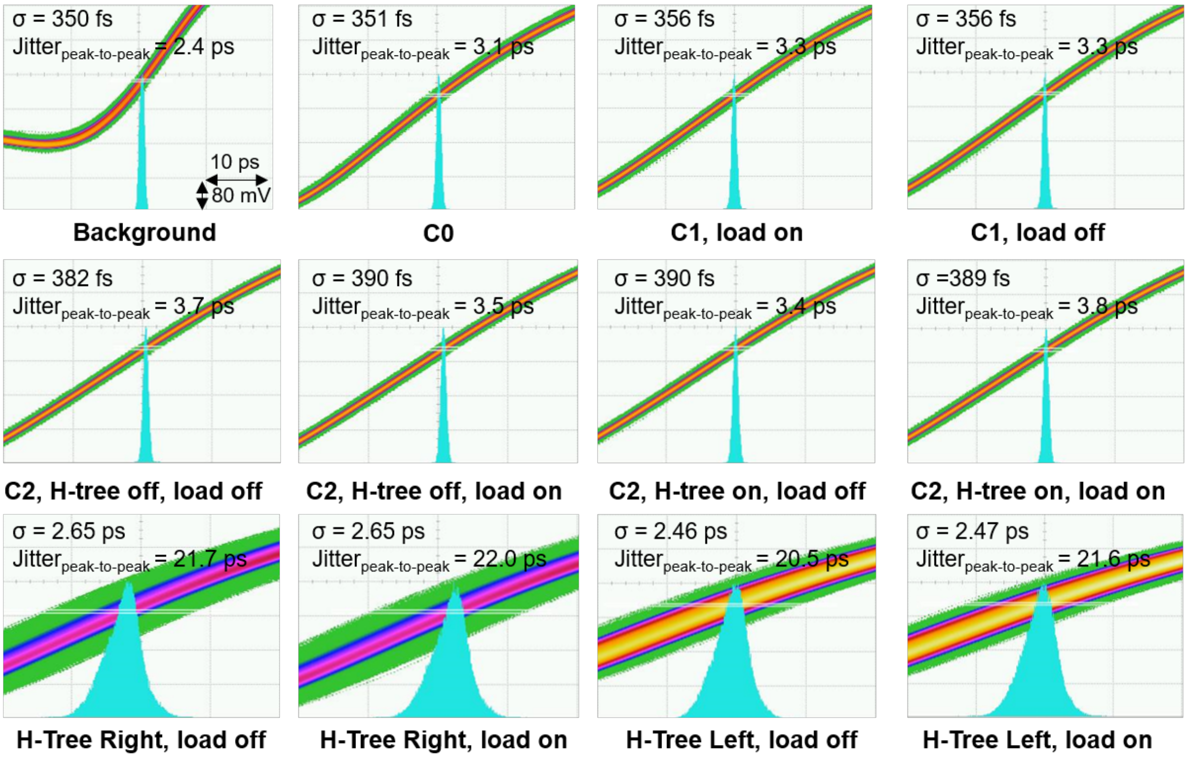

Supplementary Fig. 2. Jitter measurement results of various conditions. The measurement condition of each result is written below each figure. A 33-GHz, 128 GS/s oscilloscope is used for measurement (Keysight, UXR0334A).

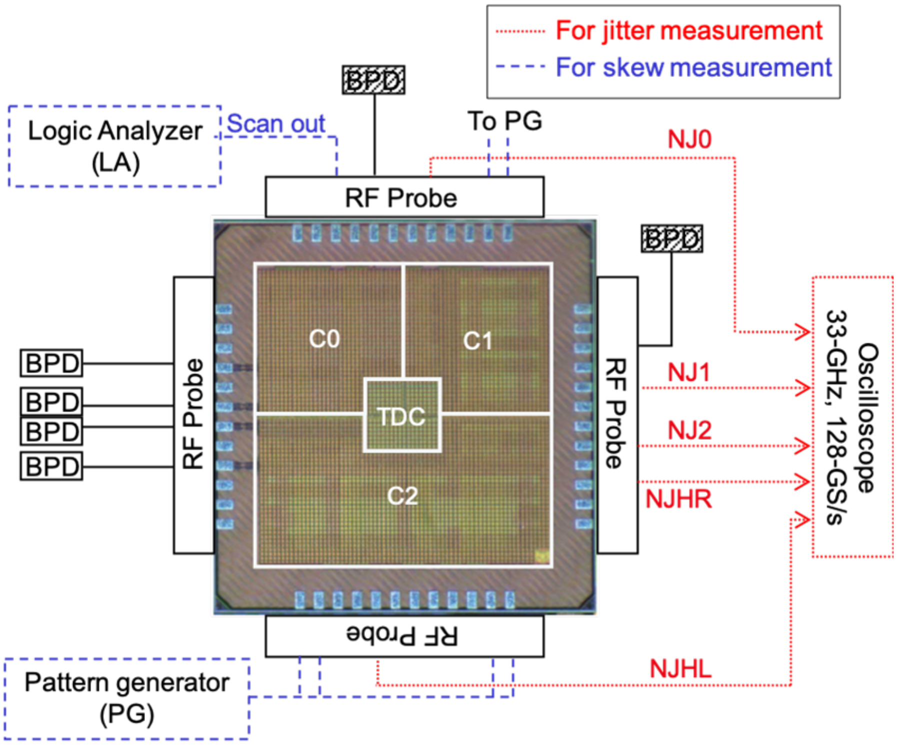

Supplementary Fig. 3. Chip test setup. High-bandwidth probes are used for high-speed chip testing. Each clock driver output is sent to the high-speed oscilloscope for clock waveform and jitter measurement. For skew measurement, a pattern generator is used to generate input, and load mode selection bit and other logic signals for TDC are applied to the chip through a scan chain. The raw TDC output is fed out from the chip using the same scan-chain, which is headed to logic analyzer.

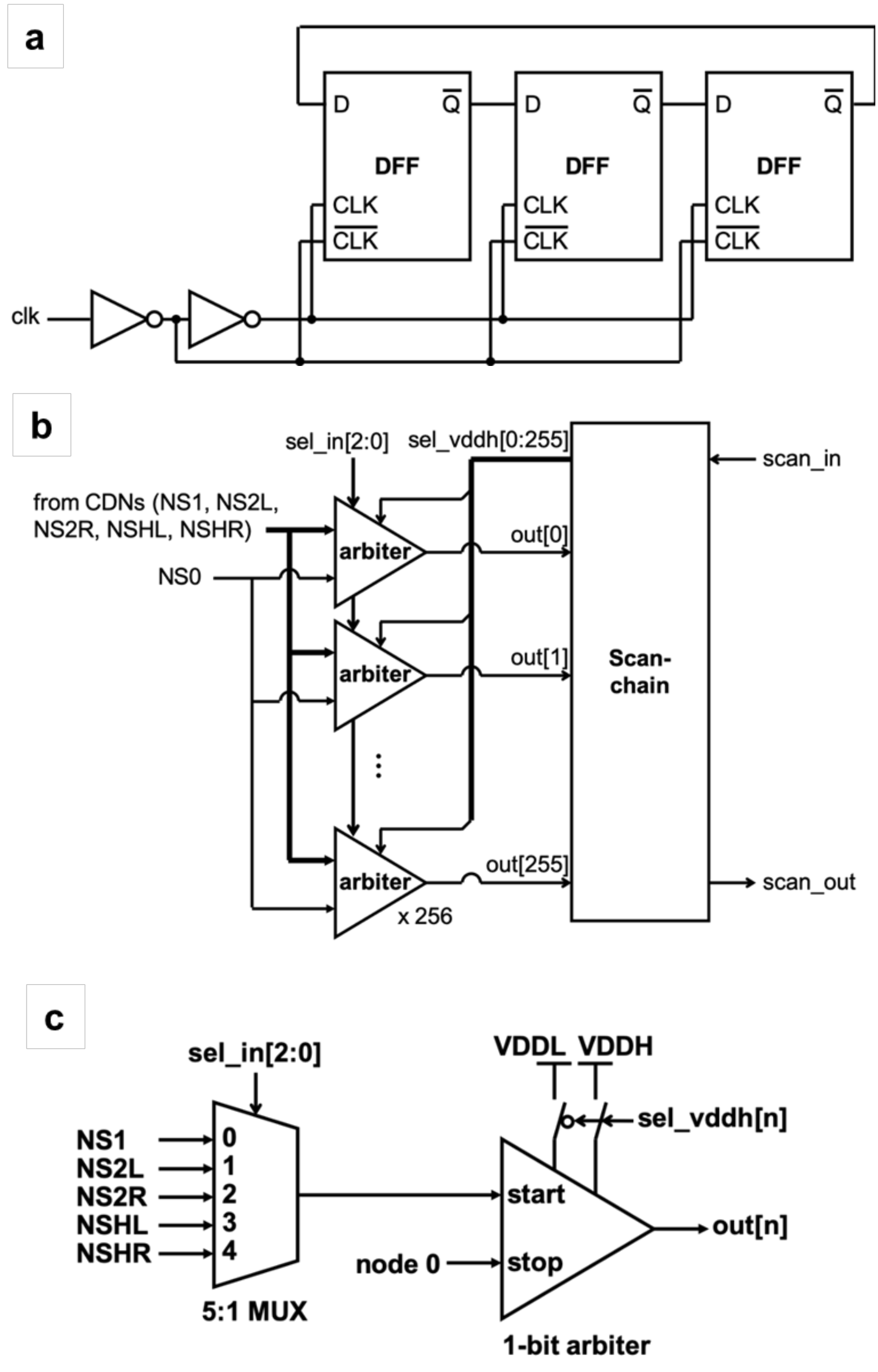

Supplementary Fig. 4. Circuit structures. a, load circuit structure. **b,** stochastic time-to-digital converter structure. **c,** 1-bit arbiter structure.

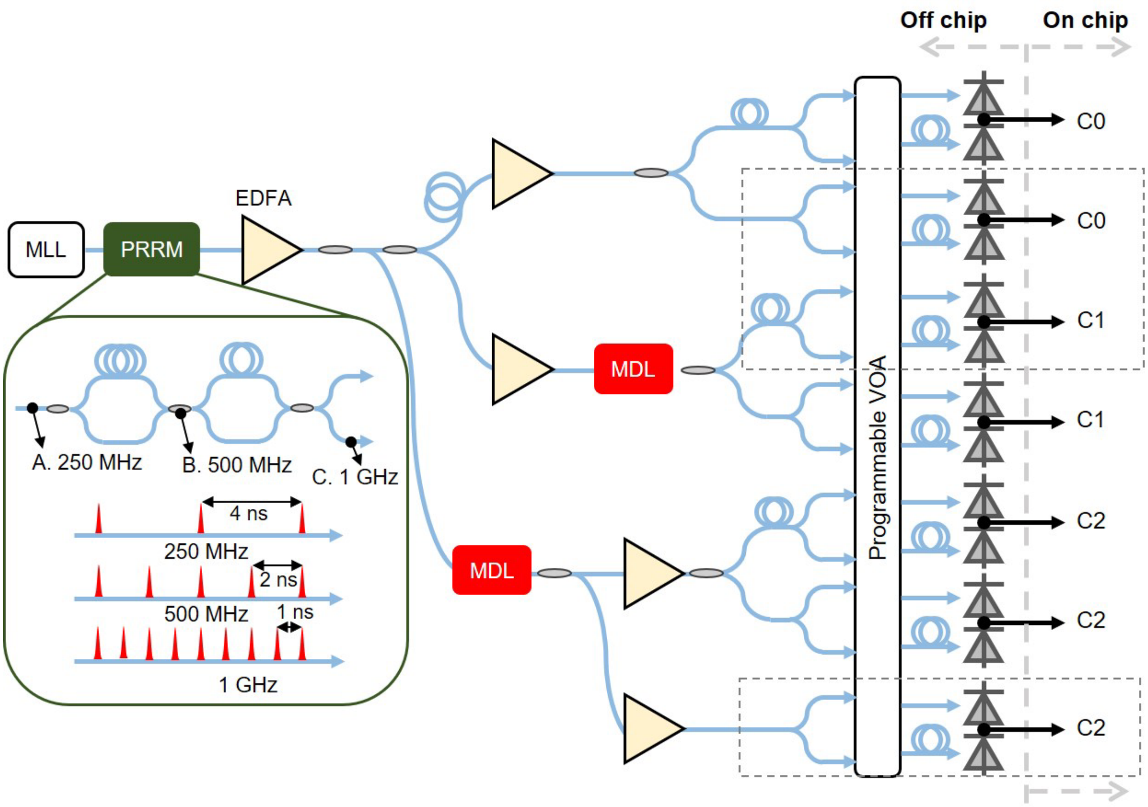

Supplementary Fig. 5. Optical setup. MLL, 250-MHz mode-locked laser; PRRM, Pulse repetition rate multiplication (Mach-Zehnder interferometer-based pulse interleaver); EDFA, Er-doped fiber amplifier; MDL, motorized delay line. MDLs are placed in front of C1, C2 domain input for TDC characterization and adaptive skew control. The photodiodes in the dashed line boxes can be used for increasing the total photocurrent pulse charge for driving higher clock rate (≥1 GHz).

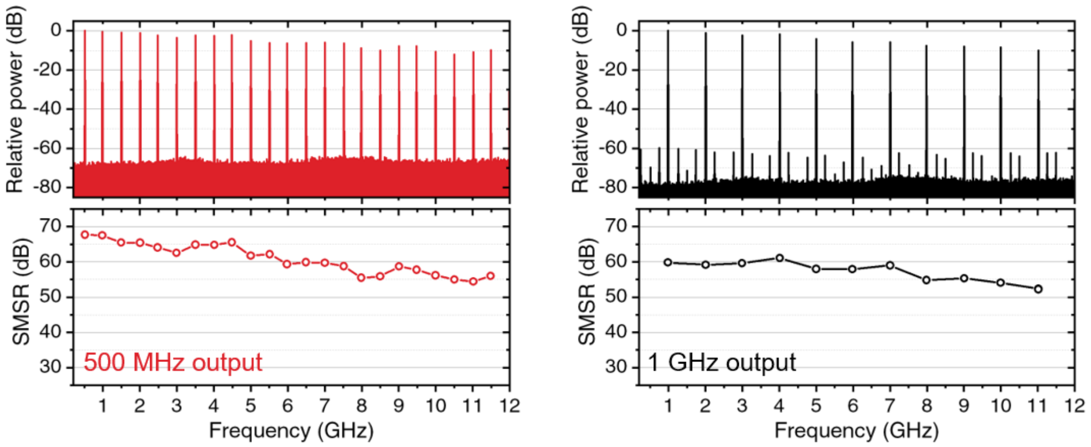

Supplementary Fig. 6. Pulse repetition rate multiplication performance. RF spectra and side mode suppression ratios (SMSRs) for repetition frequencies of 500 MHz and 1 GHz, which are the outputs from the 1st and 2nd stages of Mach-Zehnder interferometer-based pulse interleaver, respectively.

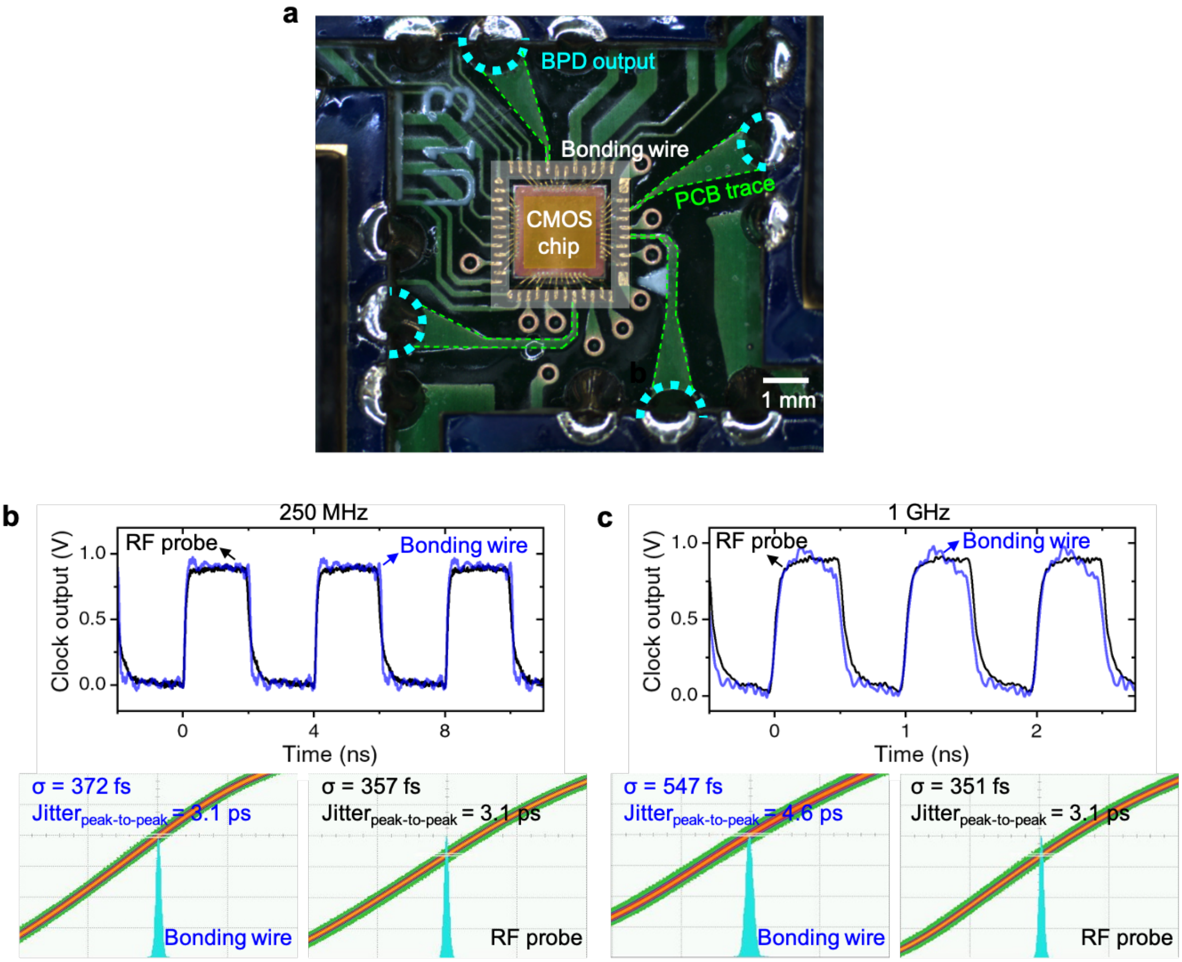

Supplementary Fig. 7. Chip-on-board (CoB) implementation of the test setup and measurement results. a, Photo of the printed circuit board (PCB). **b,** Clock waveform and timing jitter for 250-MHz repetition rate. **c,** Clock waveform and timing jitter for 1-GHz repetition rate.
